# Transcriptomic analysis of reproductive organs of pregnant mice post *toxoplasma gondii* infection reveals the potential factors that contribute to poor prognosis

**DOI:** 10.3389/fmicb.2024.1431183

**Published:** 2024-06-28

**Authors:** Meng-Ling Deng, Jun-Rong Chen, Jian-Fa Yang, Jun Ma, Fan-Fan Shu, Feng-Cai Zou, Jun-Jun He

**Affiliations:** ^1^Faculty of Animal Science and Technology, Yunnan Agricultural University, Kunming, China; ^2^Key Laboratory of Veterinary Public Health of Yunnan Province, College of Veterinary Medicine, Yunnan Agricultural University, Kunming, China

**Keywords:** *toxoplasma gondii*, transcriptome, reproductive organs, pregnant, mouse

## Abstract

*Toxoplasma gondii* is an obligate intracellular parasite of phylum Apicomplexa that poses a huge threat to pregnant hosts, and induces tragic outcomes for pregnant hosts, fetuses and newborns. However, the molecular mechanism underlying the tragic consequences caused by *T. gondii* remains to be revealed. In the present study, we applied RNA-seq to study the transcriptomic landscape of the whole reproductive organ of pregnant mice post *T. gondii* infection, aiming to reveal the key altered biological characters of reproductive organs of pregnant mice that could contribute to the tragic outcomes caused by *T. gondii* infection. The results of the present study showed that the transcriptome of reproductive organs of pregnant mice was significantly altered by *T. gondii* infection. A total of 2,598 differentially expressed genes (DEGs) were identified, including 1,449 upregulated genes and 1,149 downregulated genes. Enrichment analysis of the DEGs showed that the significantly altered features of reproductive organs of pregnant mice were excessive inflammatory responses, downregulated metabolism processes, and congenital diseases. The chemotaxis of immune cells in the reproductive organs of infected pregnant mice could also be reshaped by 19 differentially expressed chemokines and 6 differentially expressed chemokine receptors that could contribute to the damages of reproductive organ in pregnant mice. Overall, the findings of present study may help to understand the pathogenic mechanism of the acute *T. gondii* infection in reproductive organs of pregnant mice, and it could also help to improve toxoplasmosis therapeutics for pregnant individuals.

## 1 Introduction

*Toxoplasma gondii* is a worldwide zoonotic pathogen which is prevalent in all warm-blooded vertebrates, including approximately one-third of the world’s population (Galal et al., 2018). Humans acquire *T. gondii* infection through various routes, including ingestion of oocysts shed by felid definitive hosts, consumption of raw or undercooked meat contaminated with *T. gondii* tissue cysts, or vertical transmission of *T. gondii* from infected pregnant women to the growing fetuses, resulting in congenital toxoplasmosis (Mahmoudvand et al., 2015). Recent researches showed that the overall prevalence of acute *Toxoplasma* infection in pregnant women was 1.1% (Rostami et al., 2019), while the latent toxoplasmosis was estimated to be 33.8% (Rostami et al., 2020), highlighting the high risk of maternal or congenital toxoplasmosis. Toxoplasmosis has been recognized as one of the most important infectious diseases during pregnancy (Torgerson and Mastroiacovo, 2013), causing devastating consequences such as abortion, stillbirth, and physical or mental impairments in newborns (Dubey et al., 2021).

The outcomes of maternal-fetal transmission and clinical manifestations largely depend on the period of pregnancy. Generally, congenital infection rarely occurs when pregnant women infected before pregnancy or during chronic infection (Elbez-Rubinstein et al., 2009). Without treatment, the incidence of congenital infection is 10–15% when *T. gondii* is acquired in the first trimester, while the incidence of congenital infection is up to 60% when *T. gondii* is acquired in the third trimester (Wong and Remington, 1994). The female reproductive system damages caused by *T. gondii* infection, such as endometritis, ovarian dysfunction, ovarian and uterine atrophy, decrease in reproductive organs’ weight and reproductive performance, have been confirmed in both humans and animals (Senegas et al., 2009; Liu et al., 2021; Vargas-Villavicencio et al., 2022; Souza et al., 2023). Experimental studies in pregnant mice have also demonstrated that acute *T. gondii* infection can lead to atrophic and hemorrhagic uterine horns with an increased number of fetal resorptions (Senegas et al., 2009; Vargas-Villavicencio et al., 2022). All of these changes prove that there is an exact association between host reproductive organ damage, abnormal fetal growth and infertility caused by *T. gondii* infection (Brito et al., 2020; Liu et al., 2021).

Several drugs have applied to cure toxoplasmosis during pregnancy. For example, spiramycin has been the main drug of toxoplasmosis treatment for pregnant women, with the aim of preventing mother-to-child transmission (Montoya and Remington, 2008). A combination of pyrimethamine and sulfadiazine is also employed to treat congenital toxoplasmosis (Serranti et al., 2011). However, the adverse effects of drugs used to treat toxoplasmosis in pregnant women or fetuses continue to be a major challenge. Therefore, it is an urgent need to reveal the pathogenic mechanism of *T. gondii* in pregnant women. The implementation of experimental animal models of congenital toxoplasmosis that mimic the human transmission patterns and clinical manifestations provides new opportunities for impeding the progression of toxoplasmosis. Although a large number of studies have been performed to elucidate the interaction between host and *T. gondii*, the molecular features underlying the parasite-induced adverse consequences in pregnant host, which could provide novel insights into developing effective strategies to block congenital toxoplasmosis, remains to be revealed.

Transcriptomic technology, a widely used high-throughput method, is able to determine the mRNA alterations in functional cells/organs. To our knowledge, the studies that investigate gene expressional landscape of reproductive organs of the pregnant female are limited. The present study used RNA sequencing (RNA-seq) technology to analyze differential genes that could link the pathogenicity of *T. gondii* to the reproductive organs of pregnant mice.

## 2 Materials and methods

### 2.1 Ethical approval

This study was approved by the Life Science Ethics Committee of Yunnan Agricultural University with the ethical code 202301012. Pregnant Kunming mice (6–8 weeks old and pregnant for a week) were purchased from the Experimental Animal Science Department of Kunming Medical University (Kunming, China). The mice were maintained in the Animal Biosafety Level 2 (ABSL-2) laboratory at room temperature. All mice were fed with commercial diet and had access to sterile water *ad libitum.*

### 2.2 Preparation of samples

Twenty pregnant Kunming mice were randomly divided into two groups: infection group (10 mice) and non-infection control group (10 mice). After 5 days of adapting to experimental environment, each mouse of infection group was intraperitoneally injected with 200 tachyzoites of *T. gondii* PRU strain that were suspended in 100 μL sterile PBS. Control mice of non-infection group were injected with the same volume of sterile PBS via intraperitoneal injection. Seven days post-infection (DPI), all mice were humanely sacrificed by CO_2_ asphyxiation. The reproductive organs (include ovary, oviduct, uterus, vagina and vulva) of pregnant mice were collected and stored at −80°C until use.

The DNA used for verifying the infection of *T. gondii* was extracted using TIANamp Genomic DNA Kit (TIANGEN, Beijing, China) and the RNA of reproductive organs used for RNA-seq was isolated by using of the TransZol Up (TransGen Biotech, Beijing, China) according to manufacturer’s protocol. The extracted DNA and RNA were stored at −80°C until used for detection of *T. gondii* infection or RNA-seq.

Confirmation of *T. gondii* infection was performed by amplifying *T. gondii* B1 gene by using of semi-nested methods as described by Hill et al. (2006). The PCR products were examined using gel electrophoresis on a 2.0% agarose gel containing 4S Green Plus Nucleic Acid Stain (Sangon Biotech, Shanghai, China). Three mouse reproductive organs were randomly selected from control/treatment group and used for transcriptomic sequencing individually. RNA-seq library construction and RNA sequencing were carried out at Majorbio Co., Ltd.

### 2.3 Analysis of transcriptomic data

*Mus musculus* genomic file and GTF annotation file (Version: GRCm38) were downloaded from Ensembl genome database^1^ and used as reference data for transcriptomic analysis. In this study, FastQC was used for the filtering of raw reads, Hisat2 (Kim et al., 2019) was used for read mapping, StringTie (Pertea et al., 2015) was used for calculating gene expression of mice, DESeq2 software^2^ was used for identification of differentially expressed genes (DEG). The gene with Padjust (*p* value adjusted with Benjamini and Hochberg method) < 0.01 and Log2 (Fold Change) > 1 or <−1 was identified as differentially expressed gene (DEG). Principal component analysis (PCA) and global gene expression correlation analysis were analyzed by using of stats package in R. KOBAS-i (Bu et al., 2021) was applied to analyze the GO terms or pathways enriched by DEG. The genes that associated with diseases were analyzed by using of KEGG Disease option in KOBAS-i database. The enriched GO terms or pathways with Padjust <0.01 were considered significantly enriched. Chemotaxis network among chemokines, chemokine receptors and its targeted immune cells was reconstructed/predicted according to previous review (Griffith et al., 2014).

### 2.4 Data verification by using of quantitative real-time PCR

The RNA sample used for the construction of RNA-seq library was also reverse-transcribed into cDNA using TransScript^®^ Uni All-in-One First-Strand cDNA Synthesis SuperMix for qPCR Kit (TransGen Biotech, Beijing, China). Quantitative real-time PCR was performed by using of the PerfectStart^®^ Green qPCR SuperMix (TransGen Biotech, Beijing, China) and Bio-Rad CFX96. Ten genes (Acod1, CD52, Fam13a, Gbp8, H2-D1, H2-DMb2, Mgl2, Mzb1, Cpxm1 and Ubb-ps) were randomly selected to verify the data of RNA-seq, and β-actin was used as reference gene to normalize the expression of tested genes. The primers used for qRT-PCR are shown in Table 1. The qRT-PCR conditions were set as follows: denaturation at 94°C for 30 s followed by 40 cycles of denaturation at 94°C for 5 s, annealing at 60°C for 30 s. Melting curve analysis ranged from 65°C to 95°C to ensure that specific products were amplified in each qRT-PCR reaction. The relative gene expressional alteration was calculated by using of the 2^−ΔΔCt^ method (Livak and Schmittgen, 2001).

**Table 1:** Primer sequences used in the qRT-PCR experiments.

Primers	Sequence (5′ to 3′)
β-actin-F	TCACATCTGGCTGTTGGACC
β-actin-R	GATGTGGTCAGCAGGGAACA
Acod1-F	AGTGGGGCCTCATCCATCAT
Acod1-R	GCTATCAACCCTTCCCGTGG
CD52-F	ATGTCCGGCTTCGAGTCATC
CD52-R	GGGGGACATCCTGGTTCTTG
Fam13a-F	ACGTTGGCAGGTTGCTATGA
Fam13a-R	CCAGATGCCCTTGGTCTGAG
Gbp8-F	GCTCTCACACACTCCAGCAGA
Gbp8-R	GTCCACGTTTTCAGGTCTTCG
H2-D1-F	AAGGAGGGGGTTCAAGGTCTTC
H2-D1-R	GGAAGCCAGGAATTCCACCAGT
H2-DMb2-F	AGGCTTCATTGCCTCATGGT
H2-DMb2-R	TGTGTCCGAATCGATGGTGG
Mgl2-F	CTCTGGTGAACAGAAGGAGTCT
Mgl2-R	CCAATGGCTTTGACAGCTCAG
Mzb1-F	AGTGACGAGAGGCTTTGTCC
Mzb1-R	CGAAGATCTGCATTTTGACCTGT
Ubb-ps-F	TCACATCTGGCTGTTGGACC
Ubb-ps-R	GATGTGGTCAGCAGGGAACA
Cpxm1-F	GCGAGCTTGTGGTGTCCTAT
Cpxm1-R	ATCCTGCATGGCCCTATTCG

## 3 Results

### 3.1 Infection of *Toxoplasma Gondii* in mice and the summary of gene expression data

**Figure 1:**
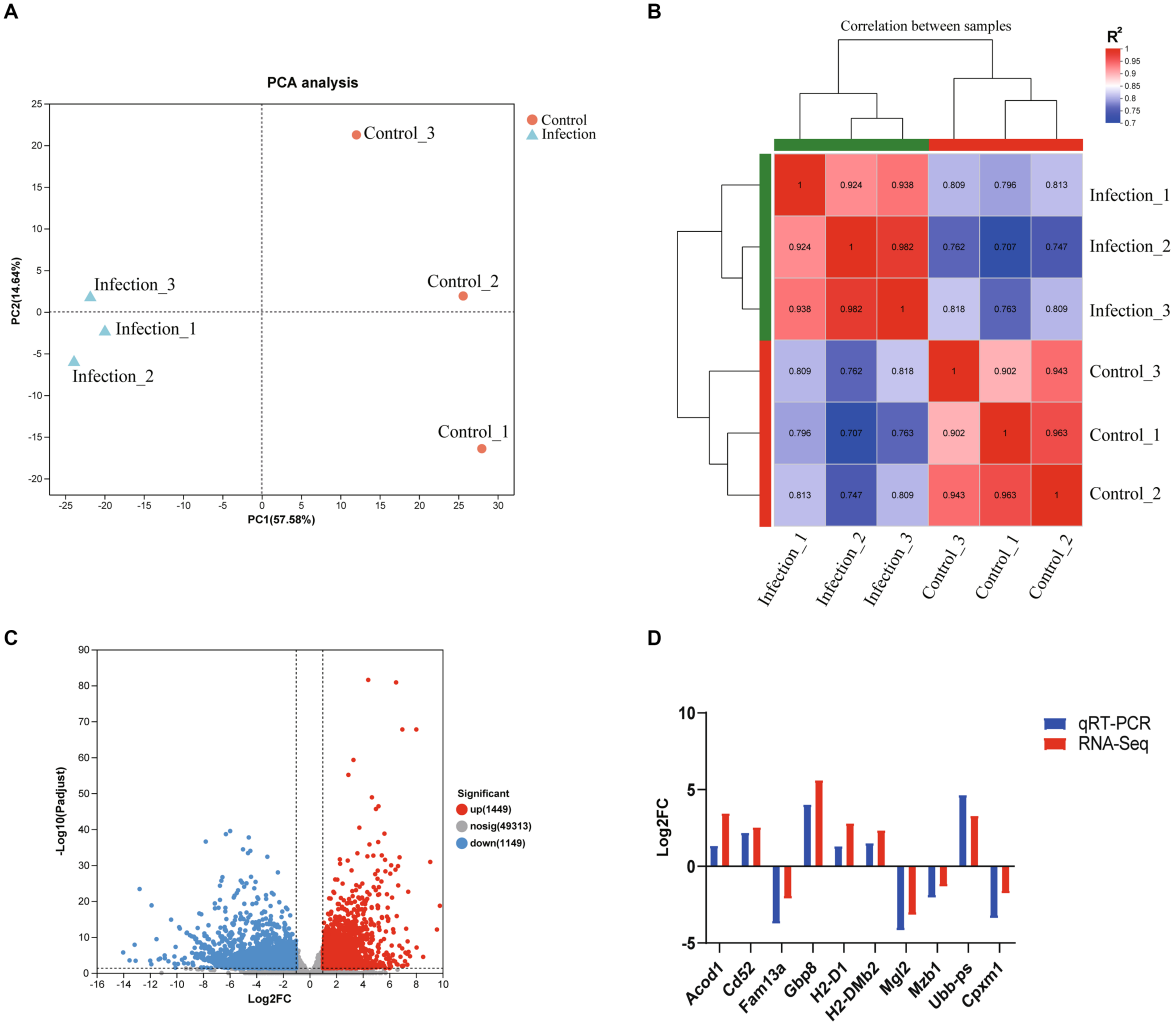
The global transcriptomic character of infected mouse reproductive organs. **(A)** PCA analysis based on global transcriptomic data. **(B)** Global correlation analysis of gene expression. **(C)** Volcano plot of differentially expressed genes. **(D)** Verification of RNA-seq data using qRT-PCR.

In this study, all sequenced samples of the infection group were positive for *T. gondii* infection, while the samples of the control group were *T. gondii* negative (Supplementary Figure S1). More than 43 million raw reads were obtained in each sample, and 90% of the raw reads met the quality evaluation criterion of Q30. 90.17–93.46% of clean reads were mapped to the mouse reference genome (Table 2). The results of principal component analysis (PCA) and global gene expression correlation analysis are shown in Figures 1A,B, respectively. In this study, a total of 2,598 DEGs were found, including 1,449 upregulated genes and 1,149 downregulated genes (Figure 1C). The DEGs identified in this study are listed in Supplementary Table S1. To verify the data obtained from RNA-seq, qRT-PCR experiment was performed, and the results of qRT-PCR experiment are shown in Figure 1D.

**Table 2:** The quality data of RNA-seq.

Sample	Raw reads	Raw bases	Clean reads	Clean bases	Error rate (%)	Q20 (%)	Q30 (%)	GC (%)	Total mapped clean reads
Control_1	47,575,178	7.18 × 10^9^	46,969,956	6.97 × 10^9^	0.0297	95.14	92.21	49.46	44,519,313 (94.78%)
Contro_2	44,085,120	6.66 × 10^9^	43,511,290	6.47 × 10^9^	0.0293	95.35	92.45	49.42	41,172,005 (94.62%)
Contro_3	46,394,452	7.01 × 10^9^	45,803,812	6.8 × 10^9^	0.0295	95.2	92.29	49.82	43,160,333 (94.23%)
Infection_1	47,405,002	7.16 × 10^9^	46,573,158	6.89 × 10^9^	0.0278	96.05	93.46	49.03	43,895,976 (94.25%)
Infection_2	43,370,022	6.55 × 10^9^	42,296,096	6.26 × 10^9^	0.0328	94.02	90.17	50.61	38,853,993 (91.86%)
Infection_3	44,122,788	6.66 × 10^9^	43,101,434	6.36 × 10^9^	0.032	94.31	90.7	50.27	39,648,338 (91.99%)

### 3.2 Summary of DEGs enrichment analysis

**Figure 2:**
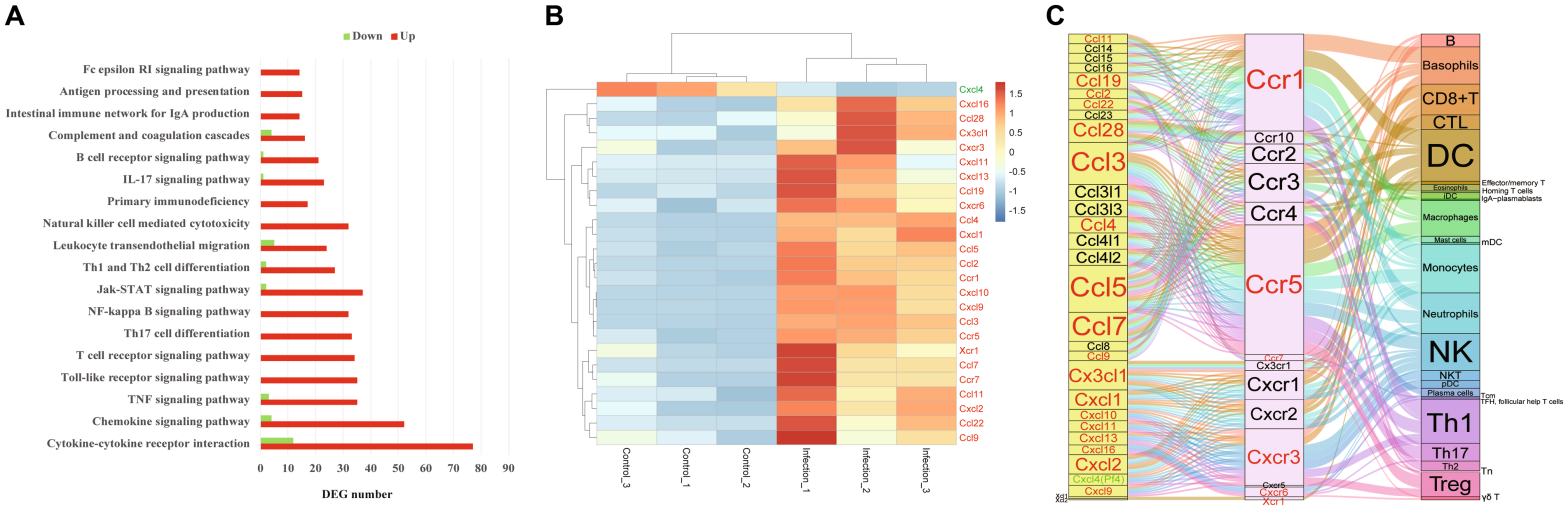
The immune pathways in female mouse reproductive organs post *T. gondii* infection. **(A)** The enriched immune pathways in female mouse reproductive organs post *T. gondii* infection. Red presents upregulation, green presents downregulation. **(B)** The heatmap of differentially expressed chemokines or chemokine receptors. Color bar presents the normalized value. **(C)** The chemotaxis network among chemokines, chemokine receptors and targeted immune cells in the infected female reproductive organs. Red character presents upregulated gene, green character presents downregulated gene.

The KOBAS-i database was applied to analyze the GO terms or pathways that were significantly enriched by DEGs. As shown in Supplementary Table S2, a total of 960 GO terms, 192 pathways and 45 diseases were significantly enriched by DEGs. In the significantly enriched GO terms, 659, 149, and 152 GO terms were classified into biological process, cellular component and molecular function, respectively. According to the Padjust value, the top 10 enriched biological processes were inflammatory response, signal transduction, immune response, neutrophil degranulation, cytokine-mediated signaling pathway, innate immune response, cellular response to lipopolysaccharide, apoptotic process, defense response to virus, and positive regulation of transcription by RNA polymerase II (Supplementary Table S2). Inflammatory response was the first biological process and 95% DEGs associated with this biological process were upregulated. According to the Padjust value, the top 10 enriched pathways were cytokine-cytokine receptor interaction, metabolic pathways, pathways in cancer, Epstein–Barr virus infection, chemokine signaling pathway, NOD-like receptor signaling pathway, measles, influenza A, viral protein interaction with cytokine and cytokine receptor, and kaposi sarcoma-associated herpesvirus infection, respectively (Supplementary Table S2). Cytokine-cytokine receptor interaction was the first enriched pathway and 86% DEGs in this pathway were upregulated (Figure 2A). Additionally, 175 GO terms and 34 pathways were only enriched by the upregulated or downregulated DEGs. Among the 45 enriched diseases, most immune related diseases were dominated by upregulated DEGs, while most congenital diseases or reproductive system diseases were dominated by downregulated DEGs.

### 3.3 The pathways associated with immune environment alterations

In the present study, a total of 18 pathways associated with immune environment were significantly enriched, including cytokine–cytokine receptor interaction, chemokine signaling pathway, TNF signaling pathway, Toll-like receptor signaling pathway, T cell receptor signaling pathway, Th17 cell differentiation, NF-kappa B signaling pathway, Jak-STAT signaling pathway, Th1 and Th2 cell differentiation, leukocyte transendothelial migration, natural killer cell mediated cytotoxicity, primary immunodeficiency, IL-17 signaling pathway, B cell receptor signaling pathway, complement and coagulation cascades, intestinal immune network for IgA production, antigen processing and presentation, and Fc epsilon RI signaling pathway. Interestingly, all DEGs were upregulated in the following pathways, including Toll-like receptor signaling pathway, T cell receptor signaling pathway, Th17 cell differentiation, NF-kappa B signaling pathway, natural killer cell mediated cytotoxicity, intestinal immune network for IgA production, antigen processing and presentation, Fc gamma R-mediated phagocytosis, and Fc epsilon RI signaling pathway (Figure 2A).

**Figure 3:**
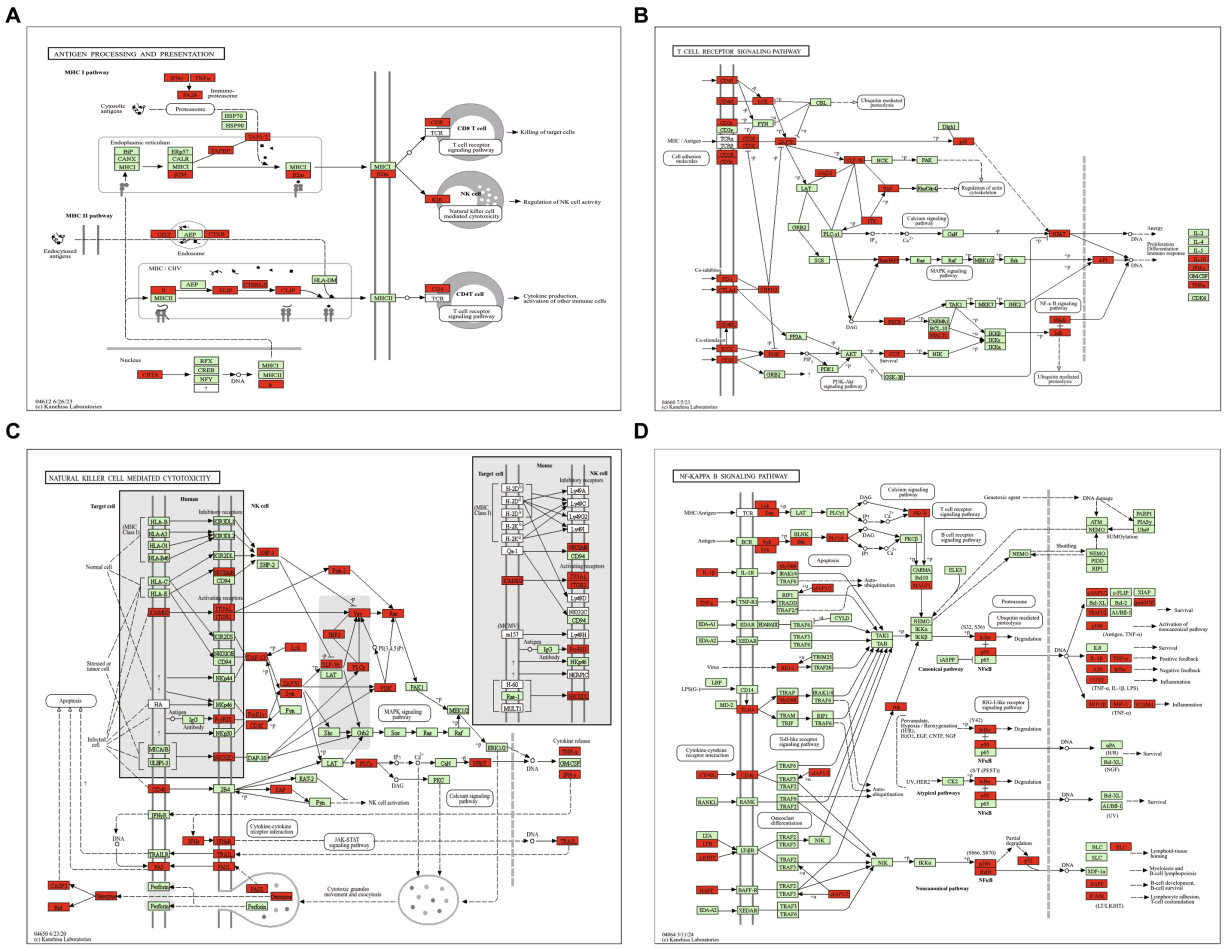
The maps of immune pathways. **(A)** Antigen processing and presentation pathway. **(B)** T cell receptor signaling pathway. **(C)** Natural killer cell mediated cytotoxicity pathway. **(D)** NF-kappa B signaling pathway. Red highlight presents upregulated DEG.

The expressions of 19 chemokines (Ccl11, Ccl19, Ccl2, Ccl22, Ccl28, Ccl3, Ccl4, Ccl5, Ccl7, Ccl9, Cx3cl1, Cxcl1, Cxcl10, Cxcl11, Cxcl13, Cxcl16, Cxcl2, Cxcl9 and Cxcl4 which also known as Pf4) and 6 chemokine receptors (Ccr1, Ccr5, Ccr7, Cxcr3, Cxcr6 and Xcr1) were significantly altered in the mouse reproductive organs post *T. gondii* infection (Figure 2B). The chemotaxis network among chemokines, chemokine receptors, and its targeted immune cells was reconstructed/predicted according to previous review (Griffith et al., 2014). As shown in Figure 2C, the 19 differentially expressed chemokines and 6 differentially expressed chemokine receptors regulate the chemotaxis of 27 immune/inflammation cells in the infected reproductive organs of pregnant mice, including dendritic cells (DC), neutrophils, monocytes, macrophages, Th1 cells, basophils, NK cells, Th17 cells, Treg cells, CTL, CD8 + T cells, mDC, B cells, Tn, Tcm, pDC, NKT cells, plasma cells, γδ T cells, Th2 cells, iDC, effector/memory T cells, eosinophils, mast cells, TFH (follicular help T cells), homing T cells and IgA-plasmablasts. The immune pathway maps of natural killer cell mediated cytotoxicity, antigen processing and presentation, NF-kappa B signaling pathway, and T cell receptor signaling pathway are shown in Figure 3.

### 3.4 The pathways associated with metabolism alterations

**Figure 4:**
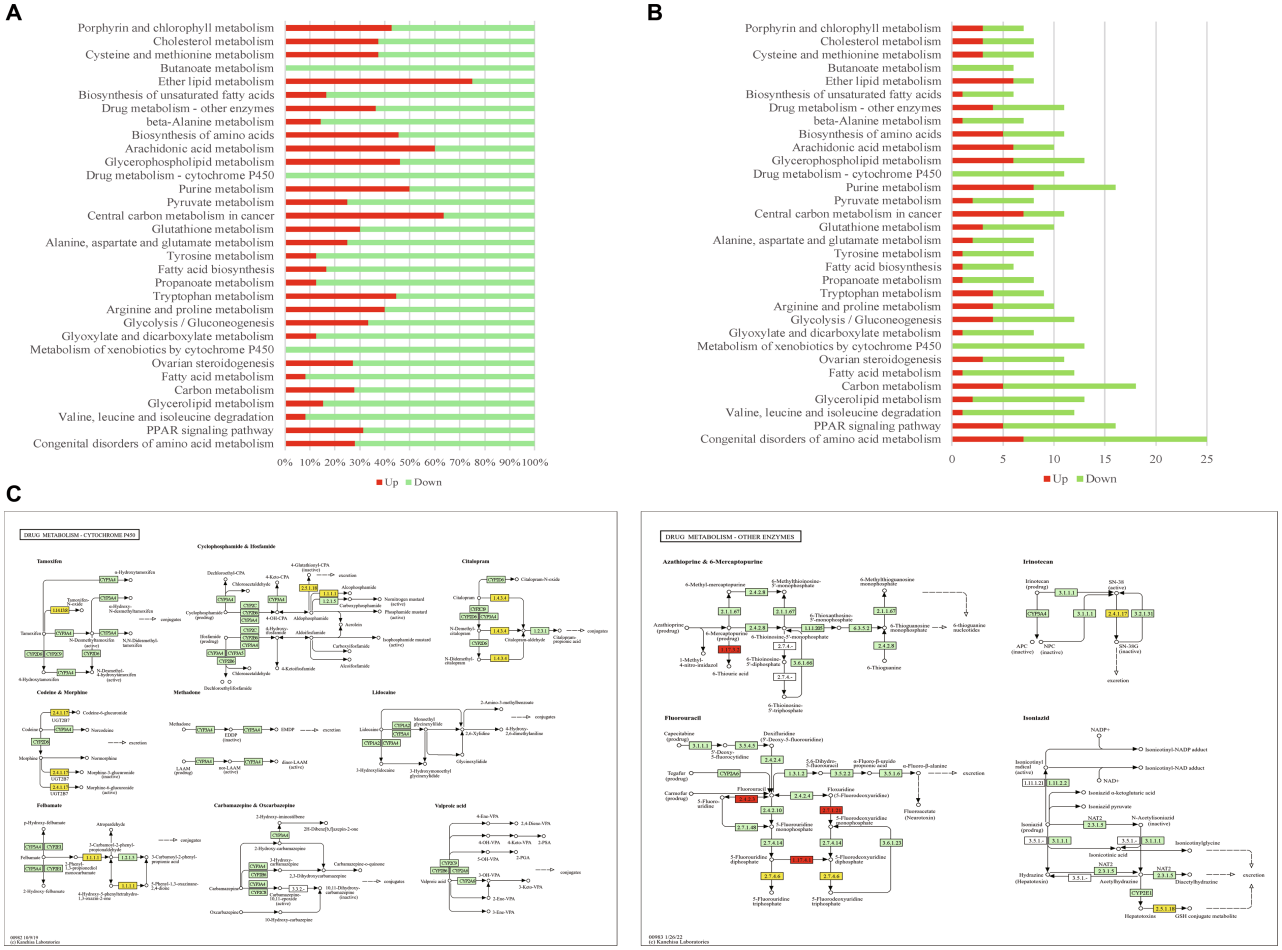
The metabolism pathways enriched by DEGs. **(A)** The percentage of upregulated/downregulated DEGs in enriched metabolism pathways. **(B)** The DEG number of enriched metabolism pathways. **(C)** The maps of drug metabolism-cytochrome P450 pathway and drug metabolism-other enzymes pathway. Red highlight presents upregulated gene, yellow highlight presents downregulated gene.

In this study, 32 metabolic pathways were significantly enriched by the DEGs of mouse reproductive organs, such as glycerolipid metabolism, fatty acid metabolism, arginine and proline metabolism, tryptophan metabolism, tyrosine metabolism, pyruvate metabolism, drug metabolism, metabolism of xenobiotics by cytochrome P450, butanoate metabolism, and congenital disorders of amino acid metabolism. As shown in Figure 4A, most of these enriched pathways were dominated by downregulated DEGs, and only 3 pathways (ether lipid metabolism, central carbon metabolism in cancer, and arachidonic acid metabolism) were dominated by upregulated DEGs. Among the 32 enriched metabolism pathways, congenital disorders of amino acid metabolism was regulated by most DEGs (Figure 4B). The alterations of drug metabolism pathway in infected reproductive organs of pregnant mice are shown in Figure 4C.

### 3.5 The alteration of disease associated genes

**Figure 5:**
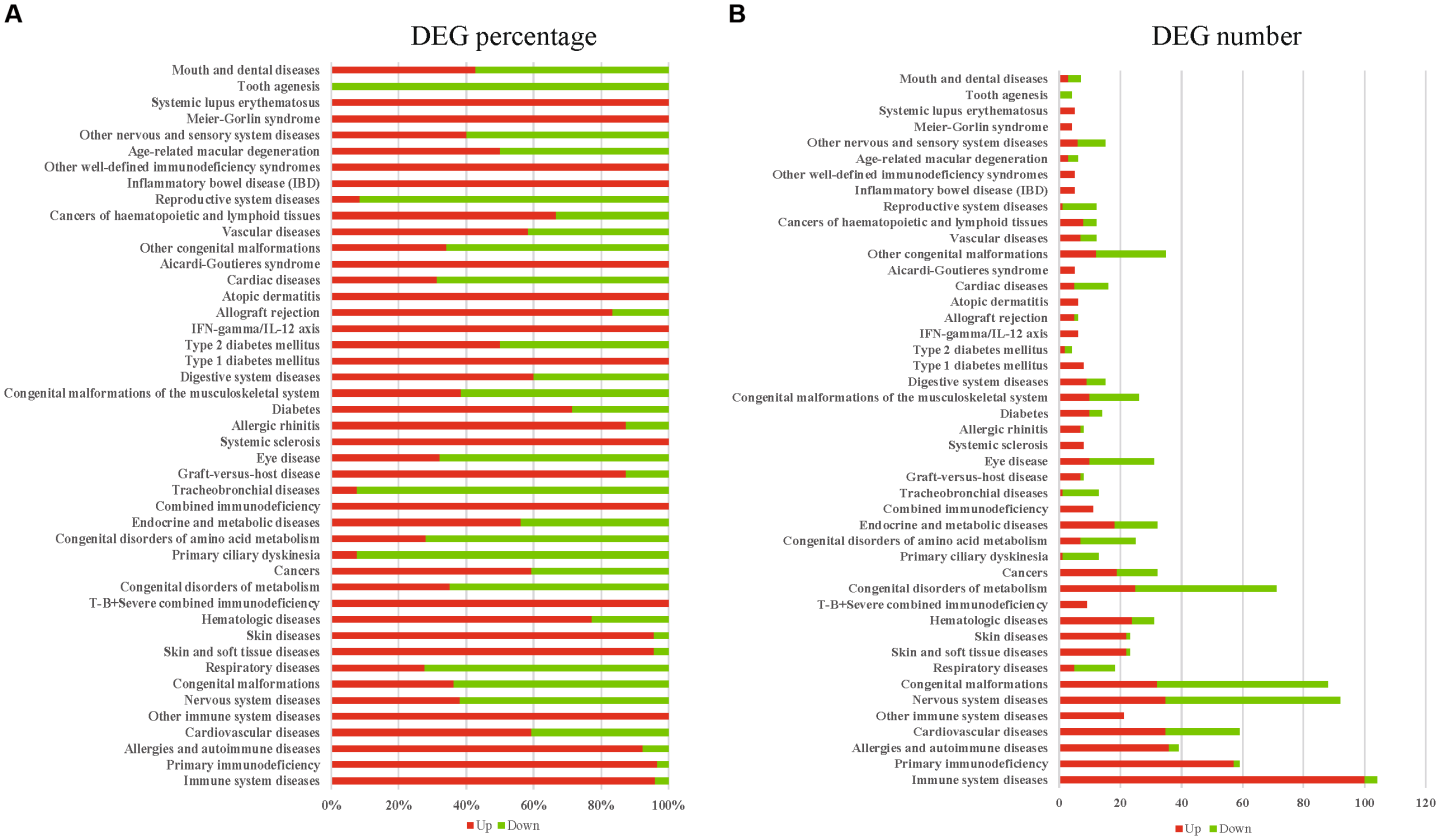
The diseases enriched by DEGs. **(A)** The upregulated or downregulated DEG percentage of enriched diseases. **(B)** The upregulated or downregulated DEG number of enriched diseases. Red presents upregulation, green presents downregulation.

In the infected reproductive organs of pregnant mice, a total of 45 diseases were significantly enriched by DEGs, including immune system diseases, primary immunodeficiency, allergies and autoimmune diseases, cardiovascular diseases, other immune system diseases, nervous system diseases, respiratory diseases, skin and soft tissue diseases, skin diseases, hematologic diseases, T-B+ severe combined immunodeficiency, cancers, primary ciliary dyskinesia, endocrine and metabolic diseases, combined immunodeficiency, tracheobronchial diseases, graft-versus-host disease, systemic sclerosis, allergic rhinitis, diabetes, digestive system diseases, type 1 diabetes mellitus, type 2 diabetes mellitus, IFN-gamma/IL-12 axis, allograft rejection, atopic dermatitis, cardiac diseases, Aicardi-Goutieres syndrome, vascular diseases, cancers of haematopoietic and lymphoid tissues, inflammatory bowel disease (IBD), other well-defined immunodeficiency syndromes, age-related macular degeneration, other nervous and sensory system diseases, Meier-Gorlin syndrome, systemic lupus erythematosus, tooth agenesis, mouth and dental diseases, eye disease, congenital malformations, congenital disorders of metabolism, congenital disorders of amino acid metabolism, congenital malformations of the musculoskeletal system, other congenital malformations, and reproductive system diseases. Most DEGs involved in infection or inflammation diseases were upregulated. However, most DEGs involved in congenital or reproductive diseases were downregulated (Figures 5A,B). The enriched diseases and their corresponding DEGs are shown in Supplementary Table S2.

## 4 Discussion

*Toxoplasma gondii* is an opportunistic pathogen. However, it is fatal for immunocompromised patients or organ transplant recipients who receive immunosuppressive medications. It is clear that the infection of *T. gondii* can cause severe damage to female reproductive organs (Khan et al., 2001; Nishimura et al., 2017), and infection of *T. gondii* in pregnant hosts could lead to neurological defects in infants and induce devastating consequences, such as abortion, malformation, and stillbirth (Dubey, 2022). *T. gondii* cysts have been found in vaginal smears (Dominguez and Giron, 1976) and cervical smear (Gomez-Chavez et al., 2019) of women, and ovarian dysfunction caused by *T. gondii* infection has also been confirmed (Khan et al., 2006). Previous study confirmed that infection of *T. gondii* can cause significant damage and transcriptomic alteration in the epididymis of male mice (Zheng et al., 2019). However, the transcriptomic or microenvironment alterations in reproductive organs of pregnant hosts post *T. gondii* infection remains to be revealed. In this study, we applied RNA-seq to study the transcriptomic alteration in the whole reproductive organ of pregnant mice post *T. gondii* infection, aiming to provide clues for studying the biological changes that could contribute to abortion, malformation, stillbirth and the other devastating consequences caused by *T. gondii* infection.

PCA and global gene expression correlation analysis are widely used methods for revealing the variation between samples. As shown in Figure 1A, the infected samples and non-infected samples were clustered to different areas of the PCA plot. Global gene expression correlation analysis showed that the transcriptomic landscapes of the same group showed a high correlation index (*R*^2^ > 0.9), while the transcriptomic landscapes between different groups had a relatively low correlation index (*R*^2^ approximately equal to 0.8) (Figure 1B). The results of global gene expression correlation analysis consistent with those of the PCA analysis, which suggested that the transcriptomic landscapes between infected reproductive organs and non-infected reproductive organs were totally different, and infection of *T. gondii* had a significant influence on the transcriptome of reproductive organs of pregnant mice. In the present study, a total of 1,449 upregulated genes and 1,149 downregulated genes were identified in the reproductive organs of infected pregnant mice (Figure 1C and Supplementary Table S1). Our qRT-PCR experiment confirmed that the results of RNA-seq are reliable (Figure 1D).

We wondered the primary biological functions of DEGs, GO and pathway enrichment analyses were performed. In the present study, a total of 960 GO terms were significantly enriched (Supplementary Table S2). Interestingly, most of the top 10 enriched biological processes were inflammatory/immune response related and dominated by upregulated DEGs, such as inflammatory response, immune response, neutrophil degranulation, cytokine-mediated signaling pathway, innate immune response, and defense response to virus (Supplementary Table S2). It is well known that immune/inflammatory responses of hosts are closely associated with the elimination of *T. gondii*. However, excessive inflammation/immune responses are also fatal to the life of infected hosts, especially the fetus of pregnant hosts (Savion et al., 2002; Schreiner and Liesenfeld, 2009). Maternal immune deregulation and adverse outcomes caused by *T. gondii* infection has been confirmed (Xu et al., 2013). In this study, 137 DEGs were enriched in inflammatory response (GO term: 0006954), and 124 of the 137 DEGs were upregulated, which suggested that strong/excessive inflammatory responses were induced in the reproductive organs of infected pregnant mice. Consistent with the results of GO enrichment, the cytokine-cytokine receptor interaction pathway, which plays an important role in reshaping the immune microenvironment, was the top pathway enriched by DEGs.

It is clear that alterations in immune cell composition will disrupt the balance of immune microenvironment and then contribute to adverse pregnancy outcomes (Gomez-Chavez et al., 2020). As shown in Figure 2A, the expressions of 89 genes of cytokine-cytokine receptor interaction pathway were altered in reproductive organs of pregnant mice post *T. gondii* infection, and 77 of the 89 DEGs were significantly upregulated, especially the chemokines and chemokine receptors. In the present study, a total of 19 chemokines and 6 chemokine receptors differentially expressed in the infected reproductive organs of pregnant mice. Among the 19 chemokines and 6 chemokine receptors, only one chemokine (Cxcl4) was downregulated (Figure 2B). Chemokines or chemokine receptors of cytokine-cytokine receptor interaction pathway is in charge of recruiting immune cells and reshapes the immune microenvironment of infected organs. According to the chemotaxis network of immune cells described in previous review (Griffith et al., 2014), in the present study, we found that the chemotaxis of 27 immune cells could be increased in infected reproductive organs of pregnant mice by the upregulated chemokines or chemokine receptors. The top 5 immune cells regulated by most differential chemokines and chemokine receptors were monocytes, DC, Th1 cells, neutrophils and NK cells (Figure 2C).

Monocytes can be recruited to sites of infection and differentiate into macrophages and DC to control *T. gondii* infection. Also, monocytes produce IL-1β to promote production of IFN-γ which is essential for the control of *T. gondii* (Hunter et al., 1995). However, *T. gondii*-infected monocytes are capable to adhere to placental syncytiotrophoblasts and result in destruction of the trophoblasts though TNF-α-dependent apoptosis (Juliano et al., 2006). It is well known that DC bridge the innate and adaptive immunity and are essential immune cells for the control of *T. gondii* in infected hosts (Cohen and Denkers, 2015), Th1 cells are major producers of IFN-γ (Sasai et al., 2018), NK cells have been important immune cells for the control of acute *T. gondii* infection (Gigley, 2016). However, the uncontrollable Th1 cells and NK cells are also effector cells that cause various pregnancy disorders or pregnancy loss (Veljkovic Vujaklija et al., 2012; Wang et al., 2020). In the present study, we found that the upregulated Ccr1, Ccr5, and Cxcr3 interacted with most differential chemokines and recruit several immune cells, such as monocytes, DC, Th1 cells and NK cells. Increased susceptibility to *T. gondii* infection has been confirmed in mice lacking the chemokine receptor Ccr1 (Khan et al., 2001). Ccr5 is essential for the trafficking of immune cells and critical for host survival post *T. gondii* infection (Khan et al., 2006). However, overproduction of Ccr5 is also a critical factor that contributes to interruption of early pregnancy in mice post *T. gondii* infection (Nishimura et al., 2017). Beside of Ccr5, the roles of Cxcr3 in *T. gondii* associated immunopathology in pregnant mice have also been confirmed (Nishida et al., 2021). Ccr5 interacts with a large number of chemokines, including Ccl5 and Ccl3. In the present study, the Ccl5, Ccl3, and Ccl7 were the top 3 differentially expressed chemokines that interacted with most chemokine receptors, regulating the chemotaxis of basophils, CD8 + T cells, CTL, DC, macrophages, monocytes, neutrophils, NK cells, Th1 cells, Th17 cells, Treg cells, B cells, Effector/memory T cells, eosinophils, iDC, mast cells, plasma cells, and Th2 cells. So, it is likely that the upregulated inflammatory genes, such as Ccl5, Ccl3, Ccl7, Ccr5, and Cxcr3, could be the key factors that cause mouse reproductive organ damage, and then contribute to the tragic pregnancy outcomes of toxoplasmosis.

It is clear that cross-presentation of antigens is mainly carried out by DC, and the antigen presentation is essential for initiating CD8+ T cell responses (Joffre et al., 2012), which can mediate T cells and NK cells to control *T. gondii* infection (Dupont et al., 2012). In the present study, we found that all DEGs of antigen processing and presentation pathway were upregulated in the reproductive organs of pregnant mice post *T. gondii* infection (Figure 3A). Robust Th1 and cytotoxic responses have been confirmed in the hosts with *T. gondii* infection (Poncet et al., 2019). However, uncontrollable T cell receptor signaling pathway and natural killer cell mediated cytotoxicity pathway are detrimental to health and pregnancy (Boyson et al., 2006; Rudd, 2010). In the present study, the DEGs of T cell receptor signaling pathway (Figure 3B) and natural killer cell mediated cytotoxicity were all upregulated (Figure 3C), suggesting the robust cytotoxic responses that are detrimental to pregnancy could occur in the reproductive organs of pregnant mice post *T. gondii* infection.

Beside of T cell receptor signaling pathway and natural killer cell mediated cytotoxicity pathway, we also found that all DEGs of NF-kappa B signaling pathway were upregulated in reproductive organs of pregnant mice post *T. gondii* infection (Figure 3D). NF-kappa B signaling pathway regulates transcription of more than 400 genes, including the genes involved in chemotaxis of immune cells, immune receptors, antigen presentation, apoptosis, as well as cell growth. It is an important pathway for the control of *T. gondii* infection (Caamano et al., 2000). However, unnormal activation of this pathway in pregnant animals can induce premature termination of pregnancy and the other bad outcomes for the mother and the fetus (Gomez-Chavez et al., 2021). So, strong immune/inflammatory environment in reproductive organs of pregnant hosts is a “double-edged sword” which can control the infection of *T. gondii*, however, it can also cause tragic outcomes for pregnant hosts.

Another factor that influences the pregnancy outcomes is the alteration of metabolism processes. In the present study, we found that the infection of *T. gondii* in reproductive organs of pregnant mice had a significant influence on metabolism pathways, such as tyrosine metabolism, pyruvate metabolism, fatty acid metabolism, and congenital disorders of amino acid metabolism (Figures 4A,B). Tyrosine is the precursor of dopamine and norepinephrine that regulates cognitive performance (Jongkees et al., 2015), and it is essential for the health of pregnant animals (Kudo and Boyd, 1996). Normal transportation or metabolism of pyruvate in reproductive organs is also required for pregnant women (Huckabee et al., 1962; Nieder and Corder, 1983). Fatty acids are the most important substances for pregnant animals, and disruption of maternal-placental metabolism of fatty acids will lead to malnutrition, hypotrophy, and preterm birth of fetus (Bobinski and Mikulska, 2015). Interestingly, distinct from the enriched immune/inflammatory pathways, most of these metabolism pathways enriched in present study were dominated by downregulated DEGs. So, it is likely that the downregulation of metabolism pathways in the reproductive organs could contribute to the malignant outcomes (such as stillbirth, abortion and fetal malformation) of toxoplasmosis during pregnancy.

Drug treatments play important roles in preventing the malignant outcomes of toxoplasmosis during pregnancy. In the present study, we also found that infection of *T. gondii* had a significant influence on the drug metabolism pathways of infected reproductive organs, such as drug metabolism-cytochrome P450 pathway and drug metabolism-other enzymes pathway. Most DEGs of these drug metabolism pathways were downregulated (Figure 4A). Especially, the DEGs of the drug metabolism-cytochrome P450 pathway were all downregulated (Figure 4B). As shown in Figure 4C, the genes involved in the metabolisms of tamoxifen, codeine, morphine, felbamate, cyclophosphamide, citalopram, irinotecan, and isoniazid were significantly downregulated. Tamoxifen treatment has potentially lethal effects on pregnant mice (Ved et al., 2019) and increases the risk of fetal abnormalities (Schuurman et al., 2019). Felbamate is an antiepileptic drug that can inhibit cytochrome P450 enzyme system (Morrell, 1996), which plays an important role in the detoxification of xenobiotics, cellular metabolism and homeostasis (Manikandan and Nagini, 2018). Cyclophosphamide can cause ovarian damage, cell apoptosis, and inhibition of cell proliferation in placenta (Chen et al., 2022; Furukawa et al., 2023). Citalopram is one of the most commonly used antidepressant medications during pregnancy (Miksic et al., 2018). Adverse drug reactions can be fatal to both pregnant women and fetuses. So, a safe chemotherapy to combat toxoplasmosis in pregnant hosts should base on the metabolic alteration of pregnant animals. Unfortunately, the information about the drug metabolism in pregnant hosts with *T. gondii* infection remains limited. The expressional alterations of drug metabolic genes in mouse reproductive organs imply us that we should pay more attention to the usage of tamoxifen, codeine, morphine, felbamate, cyclophosphamide, citalopram, irinotecan, and isoniazid in pregnant hosts with toxoplasmosis.

Previous studies have confirmed that *T. gondii* has the ability to regulate apoptosis process of hosts (Mammari et al., 2019), while abnormal apoptosis in the reproductive organs usually associated with pregnancy loss (Savion et al., 2002). In the present study, apoptosis pathway was enriched by 38 DEGs, including 37 upregulated DEGs and 1 downregulated DEG, suggesting that the apoptosis pathway was upregulated in mouse reproductive organs post *T. gondii* infection. The results of present study are consistent with a previous report which showed that strong apoptosis is found in placental trophoblasts post *T. gondii* infection (Shan et al., 2018). In addition, necroptosis was also significantly enriched by 33 DEGs of mouse reproductive organs post *T. gondii* infection, and all DEGs of necroptosis pathway were upregulated (Supplementary Table S2). Abnormal apoptosis and necroptosis in reproductive tissues can result in the abortion of embryos (Shan et al., 2018). It is likely that the abnormal apoptosis and necroptosis processes in mouse reproductive organs could associate with the abortion or stillbirth caused by *T. gondii* infection.

Ocular toxoplasmosis has been widely confirmed (Goh et al., 2023). In the present study, eye disease was significantly enriched by using of KOBAS-i with disease option, and most of the DEGs associated with eye disease were downregulated (Figures 5A,B). The expressional alteration of genes involved in eye disease could be novel clues for dissecting the mechanism that induces congenital eye diseases. Beside of eye disease, we found that reproductive system diseases or congenital diseases were also enriched by the downregulated DEGs, such as congenital malformations, congenital disorders of metabolism, congenital disorders of amino acid metabolism, congenital malformations of the musculoskeletal system, and other congenital malformation pathways. One limit of the present study is that we did not sequence the transcriptome of different reproductive tissues individually. Although we lose the gene expressional data of different reproductive tissues, common DEGs of female reproductive organs post *T. gondii* infection can be revealed, and then we can dissect the common features of female reproductive organs post *T. gondii* infection. The results of the present study provide novel data or clues for further investigating the pathogenic mechanism of *T. gondii* in pregnant hosts.

## 5 Conclusion

Infection of *T. gondii* posts a huge threat to pregnant hosts and causes severe damage to their reproductive organs. In the present study, we applied RNA-seq technology to study the transcriptomic landscape of reproductive organs of pregnant mice post *T. gondii* infection. Our transcriptomic results showed that the infection of *T. gondii* can cause significant transcriptomic alteration in the reproductive organs of pregnant mice. A total of 2,598 DEGs were identified in this study. Inflammation responses, metabolism, congenital malformations and reproductive system diseases were significantly enriched by the DEGs. One interesting finding was that the inflammation responses in reproductive organs were primarily regulated by upregulated DEGs, and the immune microenvironment could be reshaped by differentially expressed chemokines, chemokine receptors and the other immune related genes. However, most of the DEGs associated with metabolism, congenital malformations or reproductive system diseases were downregulated. The results of present study provide novel clues for dissecting pathogenic mechanism of *T. gondii* in pregnant hosts, and reveal several new transcriptomic characters of reproductive organs of pregnant hosts post *T. gondii* infection.

## Data availability statement

The datasets presented in this study can be found in online repositories. The names of the repository/repositories and accession number(s) can be found in the article/Supplementary material.

## Ethics statement

The animal study was approved by Life Science Ethics Committee of Yunnan Agricultural University. The study was conducted in accordance with the local legislation and institutional requirements.

## Author contributions

M-LD: Data curation, Formal analysis, Investigation, Methodology, Visualization, Writing – original draft. J-RC: Data curation, Formal analysis, Investigation, Methodology, Writing – original draft. J-FY: Investigation, Methodology, Software, Writing – original draft. JM: Data curation, Formal analysis, Investigation, Methodology, Software, Writing – original draft. F-FS: Investigation, Methodology, Visualization, Writing – original draft. F-CZ: Data curation, Funding acquisition, Methodology, Project administration, Resources, Supervision, Writing – review & editing. J-JH: Conceptualization, Investigation, Methodology, Supervision, Visualization, Writing – review & editing.

## Footnotes

^1^http://asia.ensembl.org/Mus_musculus/Info/Index

^2^https://bioconductor.org/packages/release/bioc/html/DESeq2.html
