# Supplementary figures and images for: Transcriptomic analysis of reproductive organs of pregnant mice post toxoplasma gondii infection reveals the potential factors that contribute to poor prognosis
Source: Front Microbiol. 2024 Jun 28;15:1431183. doi: 10.3389/fmicb.2024.1431183 (PMC11239361; doi:10.3389/fmicb.2024.1431183)

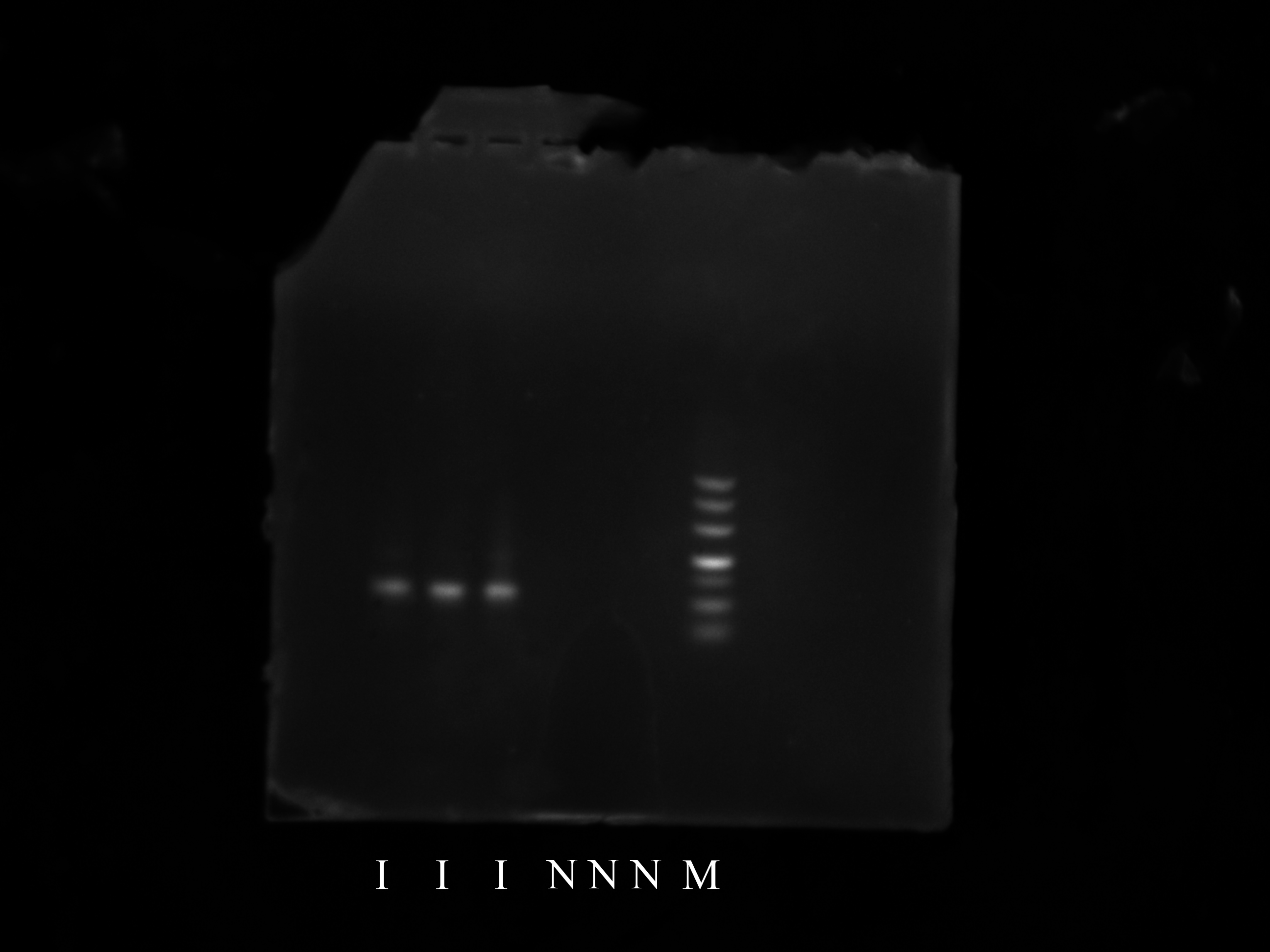

Supplement: Supplementary file 1 [file image_1.tif]
